# An End-to-End Reliability Framework of the Internet of Things

**DOI:** 10.3390/s20092439

**Published:** 2020-04-25

**Authors:** Kamal Azghiou, Manal El Mouhib, Mohammed-Amine Koulali, Abdelhamid Benali

**Affiliations:** 1 EI Research Team, École Nationale des Sciences Appliquées d’Oujda (ENSAO), Université Mohammed Premier (UMP), 60000 Oujda, Morocco; a.benali@ump.ac.ma; 2 EDER Research Team, Faculté des Sciences d’Oujda (FSO), Université Mohammed Premier (UMP), 60000 Oujda, Morocco; m.elmouhib@ump.ac.ma; 3 MSN Research Team, École Nationale des Sciences Appliquées d’Oujda (ENSAO), Université Mohammed Premier (UMP), 60000 Oujda, Morocco; m.koulali@ump.ac.ma

**Keywords:** IoT, End-to-End IoT reliability architecture, reliability, availability, reliability block diagram, failure process, maintenance

## Abstract

The Internet of Things (IoT) paradigm feeds from many scientific and engineering fields. This involves a diversity and heterogeneity of its underlying systems. When considering End-to-End IoT systems, we can identify the emergence of new classes of problems. The best-known ones are those associated to standardization for better interoperability and compatibility of those systems, and those who gave birth of new paradigms like that of Fog Computing. Predicting the reliability of an End-to-End IoT system is a problem belonging to this category. On one hand, predicting reliability can be mandatory, most times, before the deployment stage. On another hand, it may help engineers at the design and the operational stages to establish effective maintenance policies and may provide the various stakeholders and decision-makers a means to take the relevant actions. We can find in the literature works which consider only fragments of End-to-End IoT systems such as those assessing reliability for Wireless Sensors Networks (WSN) or Cloud subsystems, to cite just a few. Some other works are specific to well-defined industries, like those targeting reliability study of E-health and Smart-Grid infrastructures. Works that aims to assess reliability for an End-to-End IoT system are remarkably rare and particularly restrained in terms of expressiveness, flexibility, and in their implementation time complexity. In this paper, we apply the Reliability Block Diagram (RBD) paradigm to set up a framework for End-to-End IoT system reliability modeling and analysis. Our contribution is four-fold: we propose an IoT network-based layered architecture, we model in depth each layer of the proposed architecture, we suggest a flow chart to deploy the proposed framework, and we perform a numerical investigation of simplified scenarios. We affirm that the proposed framework is expressive, flexible, and scalable. The numerical study reveals mission time intervals which characterize the behavior of an IoT system from the point of view of its reliability.

## 1. Introduction

The research effort in the Internet of Things (IoT) field has received increasing attention from the research community. This effort revealed unique concepts, improved existing ones, and/or reported more issues and fixes. Among others, IoT security [1,2] and protocols stacks for the IoT [3,4] were investigated. In addition, tackling IoT applications as a support to other industries such as smart-living [5,6], industry 4.0 [7,8,9], and E-health [10] remains a timely issue.

The facets cited above deal with the IoT as an evolution and not a revolution. Their argument is that we could perceive IoT as a special expansion of the current Internet [11]. Another approach aims to deal with IoT as the result of fitting several subsystems corresponding to existing technologies [12]. The advocates of this approach prioritize a separate investigation of each sub-system. This procedure neglects one of the most powerful system theory principles: synergy principle, which states: “The whole system is always better than the addition of their parts”. In other terms, treating IoT as a whole entity will give rise to new classes of problems that the paradigm of IoT interpreted as disconnected parts is missing or/ignoring. The IoT supports several industries (or verticals) [13,14,15]. One needs to take several safety measures to avoid unpredictable behavior of such systems and consequently enhance their reliability, which is a critical property of every successful system [16,17].

Reliability concerns emerge when dealing with an End-to-End IoT system as a result of their heterogeneity. Indeed, one needs to pay great attention to normalizing and implementing the communicating subsystems interfaces to insure compatibility. Physical systems are the most important part of the IoT that can lead to unpredictable failures of the entire system. As part of hardware reliability, Mathematicians and Engineers studied those systems for a long time to reduce accident rates and preserve human life [18].

Reliability theory provides several tools for investigating and predicting the behavior of systems’ failures depending on the studied system state and its environment. In this work, we will adopt the actuarial approach, thus a function F(t), describing the probability distribution function of the time to failure random variable *T* will store all the needed information about a targeted system. In this approach, we need no explicit modeling of the load and strength. It is worth mentioning that to the best of our knowledge, there is no previous work that addresses the End-to-End reliability issues for IoT. Information can flow through an IoT architecture starting from the perception layer to reach the application one and vice versa. Topology defines the paths followed by these data flows. Applications and/or actuators are the major consumers of this data.

Scientists and engineers used various actuarial models and approaches to describe and analyze the reliability of entities modeled as systems. We can point out the following qualitative approaches to analyze the reliability for a system: Failure Modes, Effects, and Criticality Analysis (FMECA), Fault Tree Analysis (FTA), Cause and Effect Diagrams (CED), and Reliability Block Diagrams (RBD). In this work, we will use the RBD paradigm because of the high complexity of the investigated system. Our fundamental assumption is that adjunction of subsystems (Black Boxes) compose any End-to-End IoT system. Starting from this IoT layered architecture, we will introduce a system model on which we will apply the RBD analysis approach to set up a framework for End-to-End reliability analysis. Our contribution is four-fold:

- First, we introduce an IoT network-based architecture. Although, several architecture proposals have been developed. They are mainly emerging from competing companies that may have an antagonist or restricted vision. Our IoT architecture proposal is resilient to such influences.
- We apply the RBD to our proposed architecture. Although reliability assessment tools such as RBD have been used in many engineering disciplines, their usage in the IoT field is still moderate. The novelty in this approach resides in extending the RBD paradigm for assessing the reliability of any component of the proposed architecture independently its architectural level and nature (software, hardware, etc.). This conceptual contribution can be useful in the design stage. We model in depth each layer of the proposed architecture. We quantify each proposed layer model by an equation or sets of equations that allow us to assess reliability for each of the architectural layers.
- To ease the deployment of the proposed reliability modeling we suggest a flow chart to use the proposed framework. The proposed modeling flow chart highlights the facts that the introduced framework can incubate most of reliability assessment techniques, and that is hierarchical by nature. Thus, our proposed framework does not suffer from the curse of dimensionality associated to state based techniques such as in Markov Chains paradigm.
- At the end, we propose simplified scenarios for numerical investigation of the proposed framework. The numerical study, reveal remarkable mission time intervals which characterize the behavior of an IoT system from the point of view of its reliability.

The paper is organized as follow: Section 2 summarizes the sate of the art. Section 3 describes the IoT ecosystem. Key concepts and terminologies related to the reliability theory and the End-to-End reliability assessment framework are detailed in Section 4. Next, we present the framework deployment flow chart in Section 5. In Section 6 we cover numerical investigation and discuss obtained results. Finally we conclude the paper and announce key perspectives.

## 2. Related Work

In [19], the reliability of Wireless Sensor Networks (WSN) using the RBD paradigm is proposed. The authors assessed reliability for different WSN routing protocols while considering the battery level as the key reliability metric. The authors of [20] used a combinatorial approach to compute the reliability of a WSN for more realistic scenarios of failures. In their work, they used more reliability metrics: processor, sensor, transceiver and battery failures.

In [21] the authors studied the reliability of switched Ethernet technology used for substation systems automation. Y. Hai et al. used RBD and Bayesian Networks in [22] to assess reliability of a wide area protection communication system. In their work, they deal with network technologies that are used in Core Network technologies such as: SDH, ATM, and they used Fiber Optics as transmission links.

The authors in [23] used RBD and Petri Netorks to model and analyze dependability of virtual cloud computing data centers containing thousands of servers. They used RBD to model clusters and servers, along with Petri Netorks for dynamic mechanisms. All the earlier cited works have in common that they deal with just a slight part of the End-to-End IoT System.

The authors in [19,20] investigated an aspect of the perception layer according to our suggested architecture. Although in [20] we can observe more realistic assumptions, both of the works provided solutions that are context dependent.

The authors in [21,22] proposed specific industry based-studies of the communication networks parts which coincide in our proposed architecture with the Access and the Core Network layers. Both of the works did not treat the problem from the Internet topology point of view used to support the IoT data transmission.

Researchers in [23] focused on the cloud facet of what we define in our architecture as a subsystem of the Middleware layer. They guarantee scalability issues using a hierarchical method, but the designed framework did not consider top level layers that we can identify in the cloud paradigm. In addition, the work [23] does not account for models of software architecture used in the cloud such as those defined to support the IoT.

T. Nguyen et al. introduced in [24] a framework to quantify availability at Infrastructure level for the IoT. Although they adopt a hierarchical modeling approach using RBD, Fault tree (FT), and Continuous Times Markov Chain (CTMC) to apply in three consecutive stages. Many obstacles face the implementation of the proposed framework for reliability assessment of an End-to-End IoT system. Indeed, in terms of expressiveness, they did not use and/or establish any IoT architecture to specify the mathematical formalism behind the proposed framework. Furthermore, the framework formalism as introduced did not depict the boundaries between the various stakeholders which can lead to time consuming, misunderstanding, regulatory and policy constraints. In addition, w.r.t flexibility, their proposed framework fixes the techniques and the tools to be used when assessing reliability of the targeted system. Finally, in terms of time complexity, fixing the number of hierarchy levels at three involves a stiffness for implementing the suggested framework. A reliability qualified practitioner or the automation tool requires running over the whole framework every time it is judging that the preceding modeling steps were not granular enough.

In our paper, we establish an End-to-End reliability framework which fixes all the above-mentioned issues. We refer in establishing our framework to an IoT architecture. Hence, engineers can use the developed framework to assess reliability for both an End-to-End IoT system and for specific subsystems. We can enumerate the following properties of the developed framework. (i) Expressiveness: we adopt an IoT architecture of reference as the foundation of the developed mathematical formalism of the proposed framework. The adopted layered architecture divides the IoT world into five longitudinal layers, and within each longitudinal layer we have transversal components. In this way, we provide to the stakeholders a subsystem view for distributing responsibilities according to the adopted architecture. Each developed subsystem has clear boundaries leading to more agility for resolving the above-mentioned issues. (ii) Flexibility: in this work we use RBD as a reliability modeling tool. When achieving an irreducible system, the framework user is free to choose what seems to him the adequate way to analyze the targeted system. (iii) Time complexity: we propose, along with the established framework, an implementation flowchart which specify that the present framework allows the user to make iteration until he achieves a satisfying level of granularity. In addition, we should notice at this level that one can run the iterations in parallel and distributed fashion because they are specific to each architectural layer.

## 3. IoT Ecosystem

**Figure 1:**
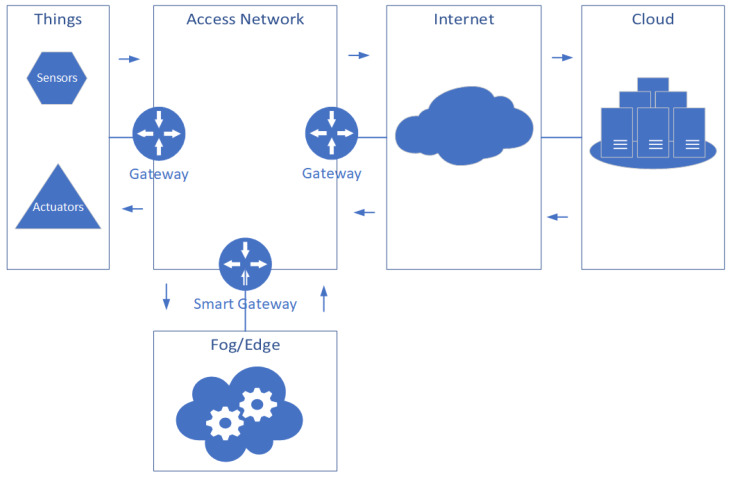
Internet of Things (IoT) ecosystem components.

The IoT comprises various entities, ranging from physical ones such as hardware and physical links for networks communication to logical ones logical ones. The latter covers operating systems, software, and services provided by the cloud. A typical IoT ecosystem is illustrated in Figure 1.

A variation of a physical phenomenon corresponds to an analog signal, which can be sensed and transformed by a sensor to a numeric one for further processing. Thus, a sensor is a building block by which the physical world and the virtual one are connected. The actuator is another building block that allows acting on the environment by receiving some control signal as an input. We distinguish two sets of network technologies, used in IoT, based on data flows. The first set is formed by technologies, which allow the exchange of data between, sensors only (or between smart objects) such as SANET, WSN. The other set is composed of network technologies that have been there for a while: 4G/3G, IP/MPLS, WiFi, and Bluetooth. Moreover, its role (the access network) is to connect the sensors domain, which is a constrained domain, to a more powerful domain in terms of computing, memory, and storage capacities [13,14]. In the IoT, we can consider two broad categories of IoT devices according to their resources. The first one is composed of those equipped with a stable power supply, decent storage, and computing capabilities. The second one is the so-called constrained devices, which use batteries as power supply. The Cloud/Fog fulfills the lack of resources by providing whatever the user needs in terms of computing, memory, and/or storage capacity [25] for the second category mentioned earlier.

## 4. System Model

In the remaining sections, we will consider an IoT system composed of several subsystems and components that collaborate in such a way to fulfill a predefined goal. Let a functional block be an element of the considered system regardless of the abstraction level being adopted: component or subsystem. The purpose of reliability engineering is to identify failures and to prevent them from occurring. A functional block failure can be defined as “the termination of its ability to perform a required function” [26].

The scientific literature comprises several methods for system reliability analysis such as Failure Modes, Effects, and Criticality Analysis (FMEA/FMECA), Fault Tree Analysis (FTA), Cause and Effect Diagrams (CED), Bayesian Belief Networks (BBN), Event Tree Analysis (ETA), and Reliability Block Diagrams (RBD) [27]. All these methods can be more or less suitable in a given context depending on the details, specificity and whether we are seeking qualitative or quantitative results. Table 1 summarizes a comparison of some widely used reliability assessment methods.

In our case, we will adopt the RBD approach, which is a success-oriented network visualizing how operating functional blocks take part in the overall system function fulfillment. A structure of a system in RBD can be represented mathematically using structure functions, which in their turn are used to calculate system reliability indices.

### 4.1. Reliability Theory Background

#### 4.1.1. Structure Function and Reliability Measures

Let *S* be a system composed of *n* components denoted *i* where i∈{1,2,3,⋯}. The state variable of component *i* is given by [27]:

(1) $$X_{i}\left(t\right)=\left\{\begin{array}{cc} 1 & \mathrm{if}\;\mathrm{component}\;i\;\mathrm{is}\;\mathrm{functioning}\;\mathrm{at}\;\mathrm{time}\;t\\ 0 & \mathrm{if}\;\mathrm{component}\;i\;\mathrm{is}\;\mathrm{in}\;a\;\mathrm{failed}\;\mathrm{state}\;\mathrm{at}\;\mathrm{time}\;t \end{array}\right.$$

where $X_{i}\left(t\right)$ is a random variable associated to the component *i*.

The state vector X(t) at time *t* is formed by n−uplets of state variables [27]:

(2) $$X\left(t\right)=(X_{1}\left(t\right),X_{2}\left(t\right),\cdots ,X_{n}\left(t\right))$$

Similarly, the state of the overall system can be described by a binary function:

(3) $$\phi \left(X\left(t\right)\right)=\phi \left(X_{1}\left(t\right),X_{2}\left(t\right),\cdots ,X_{n}\left(t\right)\right)$$

where

(4) $$\phi \left(X\left(t\right)\right)=\left\{\begin{array}{cc} 1 & \mathrm{if}\;\mathrm{component}\;i\;\mathrm{is}\;\mathrm{functioning}\;\mathrm{at}\;\mathrm{time}\;t\\ 0 & \mathrm{if}\;\mathrm{component}\;i\;\mathrm{is}\;\mathrm{in}\;a\;\mathrm{failed}\;\mathrm{state}\;\mathrm{at}\;\mathrm{time}\;t \end{array}\right.$$

ϕ(X(t)) is called the structure-function of the system or just structure.

We note the following useful probabilities interpreted respectively as the reliability of component *i* and the reliability of the system at a given time *t*

(5) $$\begin{array}{ccc} Pr\left(X_{i}\left(t\right)=1\right)=p_{i}\left(t\right) & for & i=1,2,\cdots ,n \end{array}$$

(6) $$Pr\left(\phi \left(X\left(t\right)\right)=1\right)=p_{s}\left(t\right)$$

To simplify the analysis, we assume in this paper that failures are considered, as independent events. Consequently, state variables at time t, $X_{i}\left(t\right)$ are stochastically independent. Another assumption is that the component *i* is considered as non-repairable. Non-repairable components are components that can be thrown away at the first failure. In our study, we are only interested in the first failure occurrence. In such a context, the reliability and the survivor functions are the same and take the following forms [27]:

(7) $$\begin{array}{ccc} p_{i}\left(t\right)=R_{i}\left(t\right) & for & i=1,2,\cdots ,n \end{array}$$

For the hole system *s* we have:

(8) $$p_{s}\left(t\right)=R_{s}\left(t\right)$$

#### 4.1.2. Series Structure

**Figure 2:**
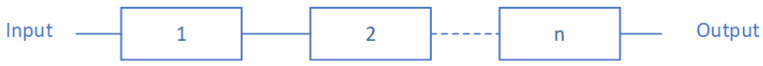
A series structure architecture according to the Reliability Block Diagram (RBD) paradigm.

A series structure is composed of components such as the failure of one of these components causes the failure of the global system. This structure is illustrated in Figure 2.

The corresponding structure function of the serial structure is given by [27]:

(9) $$\phi \left(X\left(t\right)\right)=\prod_{i=1}^{n}X_{i}\left(t\right)$$

where *i* is a component of the modeled system and X(t) is its state vector. The following equation gives the serial structure reliability [27]:

(10) $$R_{s}\left(t\right)=E(\phi \left(X\left(t\right))\right)=E\left(\prod_{i=1}^{n}X_{i}\left(t\right)\right)=\prod_{i=1}^{n}E\left(X_{i}\left(t\right)\right)=\prod_{i=1}^{n}R_{i}\left(t\right)$$

#### 4.1.3. Parallel Structure

**Figure 3:**
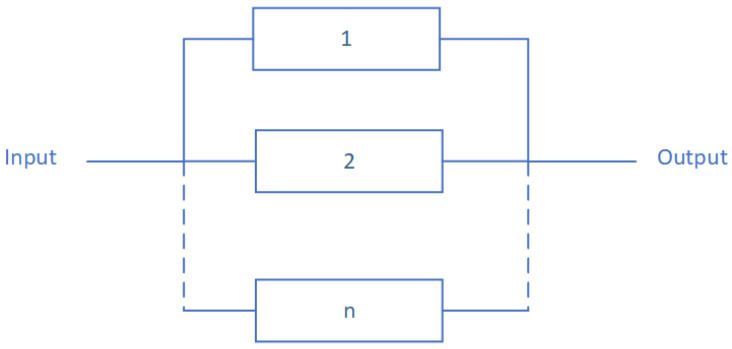
A parallel structure architecture according to the RBD paradigm.

A parallel structure organizes components such that the overall system failure happens only if all components fail. Such structure is described in Figure 3.

The corresponding structure function of parallel architecture is given by [27]:

(11) $$\phi \left(X\left(t\right)\right)=1-\prod_{i=1}^{n}\left(1-X_{i}\left(t\right)\right)$$

Parallel structures reliability is given using the following equation [27]:

(12) $$R_{s}\left(t\right)=E(\phi \left(X\left(t\right))\right)=E\left(1-\prod_{i=1}^{n}\left(1-X_{i}\left(t\right)\right)\right)=1-\prod_{i=1}^{n}E\left(\left(1-X_{i}\left(t\right)\right)\right)=1-\prod_{i=1}^{n}\left(1-R_{i}\left(t\right)\right)$$

#### 4.1.4. *k*-out-of-*n* Structure

**Figure 4:**
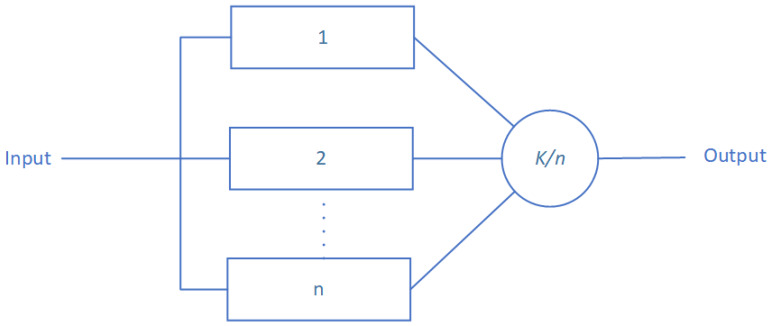
A *k*-out-of-*n* structure architecture according to the RBD paradigm.

A *k*-out-of-*n* structure of a system means that is operational if and only if at least *k* of the *n* components forming it are operational. Figure 4 describes such structure.

The corresponding structure function of a *k*-out-of-*n* system is as follows [27]:

(13) $$\phi \left(X\left(t\right)\right)=\left\{\begin{array}{ccc} 1 & if & \sum_{i=1}^{n}X_{i}\left(t\right)\geq k\\ 0 & if & \sum_{i=1}^{n}X_{i}\left(t\right)<k \end{array}\right.$$

where *i* is a component of a system and X(t) is the state vector of the system.

We assume in this case, that reliability of all the components is the same. In another way, we have $R_{i}\left(t\right)=R\left(t\right)$ for i=1,2,⋯,n, the reliability associated to *k*-out-of-*n* structure system is given by [27]:

(14) Rs(t)=∑y=knnyR(t)y×(1−R(t))n−y

### 4.2. IoT Stack as Reliability Graph

Various architecture proposals for IoT have been proposed in the literature. Some of them are introduced through consortiums or forums where stakeholders are widely known companies that participate actively to decide on the future of the IoT metamorphisms like the IoT World Forum (IoTWF) [36]. Other architecture proposals emerge from standardization bodies as ETSI and IUT [37]. We will not restrict ourselves to follow by the book a given architecture, but we will take advantage of each of them according to their relevance to the reliability aspect of the IoT systems.

#### 4.2.1. The Layered Architecture of IoT

**Figure 5:**
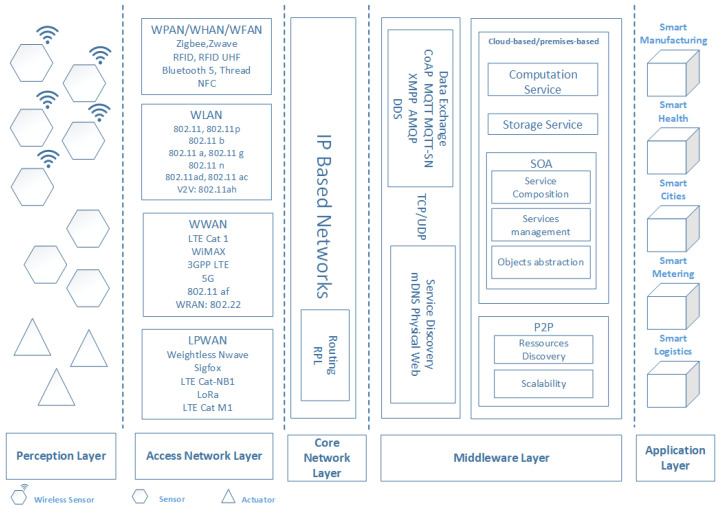
The layered architecture used for building the reliability framework of an End-to-End IoT system.

We propose the architecture illustrated in Figure 5. Our proposed architecture is composed of five layers that performs predefined tasks and functions. In the first place, we have the perception layer, which groups sensors, actuators, and smart devices denoted commonly as Things. These are isolated, and as such, they do not provide any significant application level benefit. To be of interest, “Things” need to be connected and aggregated in Sensor Networks (SNs). Wireless Sensor Networks (WSN) came to help allowing for more deployment flexibility, energy management efficiency, and applications diversity.

Moreover, most implementation cases manifest the need of other network technologies that allow clusters of Things, defined for example by WSNs, to reach the Internet. Thus, larger resources become available at worldwide scale. To do so, we add to the first layer two layers, which are respectively, the access network layer and the core network layer. Until now, compatible Things can talk to each other over the Internet. Nevertheless, incompatible ones cannot do that. Consequently, we need another layer for service diversity to abstract Things heterogeneity. The layer performing abstraction is the Middleware layer. Developers create the applications at the Application layer on top of the underlying infrastructure.

#### 4.2.2. Perception Layer Model

**Figure 6:**
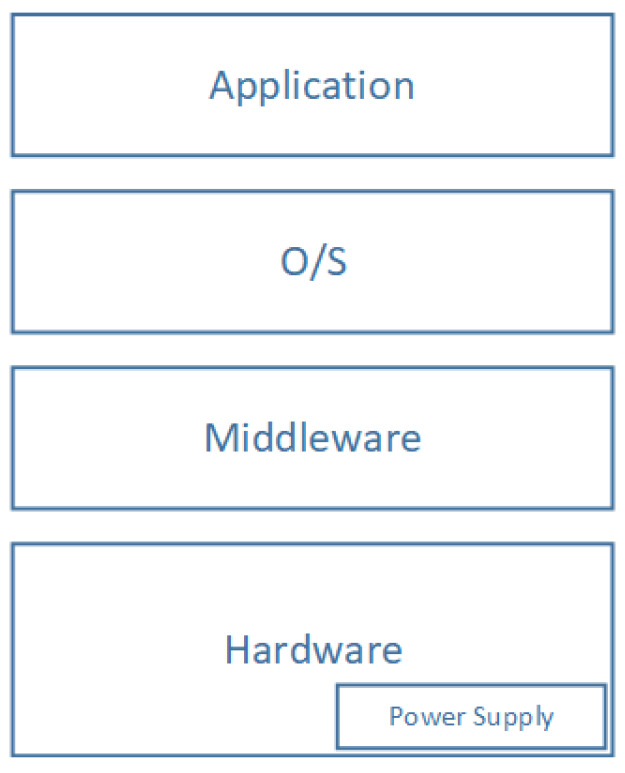
Systemic division of the Perception Layer Entity (PLE) Architecture.

The perception layer is made of Things (subsystems) that collaborate to perform a predefined mission. Such subsystems can be hardware components, operating systems, communication modules, or power supply modules. According to system theory, each subsystem is a system, which can be composed of subsystems. For instance, the hardware subsystem contains CPU, memory, and IO modules. This process can be repeated until one reaches some satisfying level of problem simplicity that can be solved easily. The adopted abstraction level depends on the context and the problem hardness. In general, an entity of perception layer can be modeled as shown in Figure 6.

**Figure 7:**
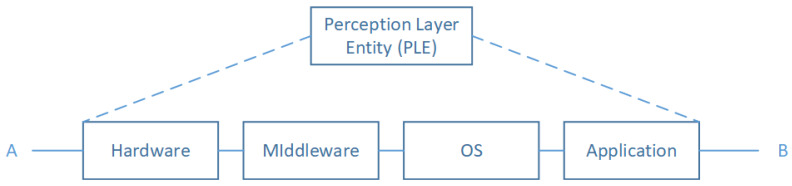
Perception Layer Entity RBD model.

For use cases not focusing on the internal structure of the Perception Layer Entities; this layer can be considered as an atomic system. A given perception layer entity modeled with the architecture in Figure 6 fails if one of the layers fails. For example, the hardware layer can be responsible for the whole system crash. In other scenarios acute power level decrease, memory overflow, and/or unsuitable environmental conditions could be the failure reason. The same effect can be observed if the software components fail to work appropriately. Our study focuses on high-level architectural failures. This gives rise to the following reliability diagram illustrated in Figure 7:

According to the proposed reliability diagram in Figure 7, we note that a Perception Layer Entity (PLE) is equivalent to four subsystems arranged according to a serial pattern. Which mean that in our adopted model if one of the subsystems fails, it will cause the whole PLE failure. As a result that we have assumed that, we will not take into account, in this work, the causes of failures. We propose to model these with a random variable, $X_{i}\left(t\right)$ denoting the subsystem *i* state. Then, the associated function structure to a PLE can be written as:

(15) $$\phi \left(X\left(t\right)\right)=\prod_{i=1}^{4}X_{i}\left(t\right)$$

where $X_{i}\left(t\right)$ for i=1,2,3,4 are the random variables describing, respectively, the state of Hardware, Middleware, OS, and application subsystems. The reliability function associated with a PLE is given by:

(16) $$R_{PLE}\left(t\right)=\prod_{i=1}^{4}R_{i}\left(t\right)$$

where $R_{i}\left(t\right)$ for i=1,2,3,4 are, respectively, the reliability function associated to Hardware, Middleware, OS, and application subsystems. The $R_{i}\left(t\right)$ function can be found by calculating the expectation value of the random variable $X_{i}\left(t\right)$ or deduced from failure rates specification tables.

#### 4.2.3. Sensors/Access Network Layer Model

This layer gives to entities of the perception layer the capability to be clustered and linked on a short-range distance according to topologies supported by the underlying network technologies. Nevertheless, in most of the cases, it seems to be not enough to be restricted by just short-range technologies. The main reason for this is that entities in the perception layer do not have enough power, memory, and computing capacities to process collected data. Thus, they seek to fulfill their needs beyond the sensors network operating range. Then, in this layer, we merge two main sets of technologies: Those responsible for connecting smart objects to form a short-range communication network of sensors and those that are not dedicated to sensors. In other words, those responsible for transporting smart objects data among other data types.

Let us consider, firstly, the sensor networks class. We assume that $PLE_{i}$ are atomic systems. That is, we consider that each $PLE_{i}$ is composed of a unique component. To communicate with each other, $PLE_{i}$ needs to establish links between them within a given area. The number of communication links depends on the context, the underlying technologies, and the adopted topology. We assume in our situation, that a system component denoted $Link_{i}$ represents all the possible links used by a given entity. The $Link_{i}$ subsystem can be studied deeply, by calculating, for instance, its corresponding structure-function considering a given scenario attributes (e.g., routing strategies). Moreover, a failure of $PLE_{i}$ does not involve, in general, the failure of the whole network. There is a threshold that has to be reached to be the case. We propose the following reliability diagram corresponding to these classes of Networks.

**Figure 8:**
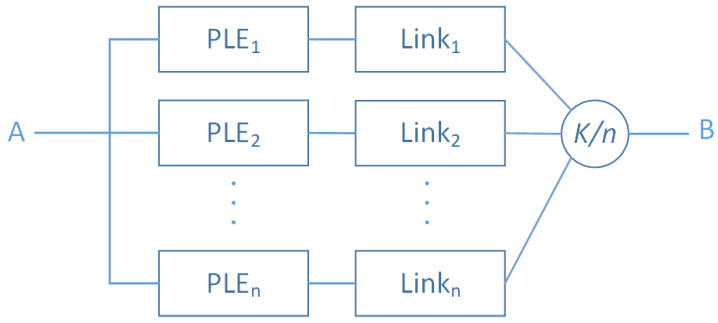
Sensors/Access Network layer entities’ RBD Model

Figure 8 illustrates the structural model of the sensor network classes in which a sensor network subsystem SN fails if and only if $k+1(PLE_{i},Link_{i})$ fail. The couple $\left(PLE_{i},Link_{i}\right)$ is a serial pattern subsystem. We assume, for simplicity, that:

(17) $$\left\{\begin{array}{ccc} R_{PLE_{i}}\left(t\right) & = & R_{PLE}\left(t\right)\\ R_{Link_{i}}\left(t\right) & = & R_{Link}\left(t\right) \end{array}\right.$$

The corresponding reliability function of such system is given, by:

(18) RSN(t)=∑y=knnyR(t)y×(1−R(t))n−y

where

(19) $$R\left(t\right)=R_{PLE}\left(t\right)\times R_{Link}\left(t\right)$$

The second part of this layer is network technologies, which are used for accessing the local area data processing sites or used to access the Internet. Their study is beyond the scope of this work for two reasons: the first one is that the context of our work does not allow such level of detail. The second reason is that some works are already focused on the reliability assessments of such network technologies, like in [38]. In our case, a high-level view of such systems seems to be enough to construct the corresponding reliability models. We observe in this part of the layer, that a Network Technology $NT_{i}$ can be used alone to drive data into processing sites or used besides other $NT_{j}$ with i≠j to this end. Thus, the communication process can be done based on gateways located at the $NT_{i}$ borders. In terms of RBD, two types of Access Network (AN) system components can represent these interactions: $NT_{i}$ component stands for Network Technology *i* where *i* is an integer that indexes a set $NT=\{NT_{i}|i\;\mathrm{indexes}\;a\;\mathrm{possible}\;\mathrm{access}\;\mathrm{network}\;\mathrm{technology}\}$. Gateway component can be considered as a simple component if it is composed of a unique physical gateway or can be a subsystem that needs to be specified with a structure-function if it is made from several physical gateways. We stress that software components embedded in a gateway are omitted in this scope.

**Figure 9:**
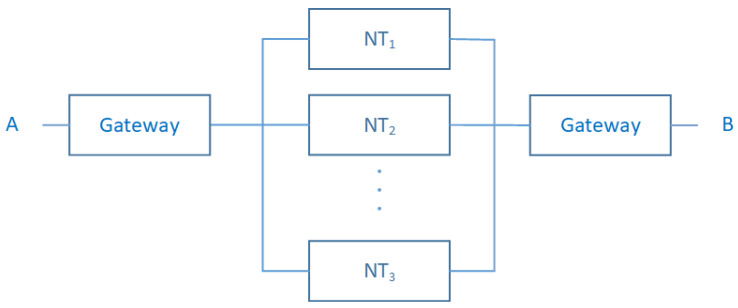
Access Network RBD Model.

The model in Figure 9, introduces the failure mechanisms that can take place in the Access Network layer at a high-level view. A subsystem of ${\left\{NT_{i}\right\}}_{i=1}^{n}$ parallel network technologies is mounted in serial pattern with gateway components. The meaning of this structure is as follows: The whole system stops to work if the entire implemented $NT_{i}$ crash and if one of the gateway components is no longer functioning. Structure and reliability functions can be written as:

(20) $$\phi_{AN}\left(X_{AN}\left(t\right)\right)=X_{Gate}\left(t\right)\times \left(1-\prod_{i=1}^{n}\left(1-X_{NT_{i}}\left(t\right)\right)\right)\times X_{Gate}\left(t\right)$$

where

- $\phi_{AN}$: Structure function of the Access Network.
- $X_{AN}\left(t\right)$: State vector of the Access Networks.
- $X_{Gate}\left(t\right)$: State vector of the Gateway subsystem.
- $X_{NT_{i}}\left(t\right)$: State vector of the implemented Network Technology $NT_{i}$.

(21) $$R_{AN}\left(t\right)=R_{Gate}\left(t\right)\times \left(1-\prod_{i=1}^{n}\left(1-R_{NT_{i}}\left(t\right)\right)\right)\times R_{Gate}\left(t\right)$$

where

- $R_{AN}\left(t\right)$: Reliability function of Access Network system.
- $R_{Gate}\left(t\right)$: Reliability function of the Gateway subsystem.
- $R_{NT_{i}}\left(t\right)$: Reliability function of the implemented Network Technology $NT_{i}$.

Let

(22) $$R_{NT}\left(t\right)=1-\prod_{i=1}^{n}\left(1-R_{NT_{i}}\left(t\right)\right)$$

Equation (21) can be re-expressed as follows:

(23) $$R_{AN}\left(t\right)=R_{Gate}\left(t\right)\times R_{NT}\left(t\right)\times R_{Gate}\left(t\right)$$

where $R_{NT}\left(t\right)$ is the reliability function of the deployed Network Technology subsystem in the modeled Access Network.

#### 4.2.4. Core Network Layer Model

**Figure 10:**
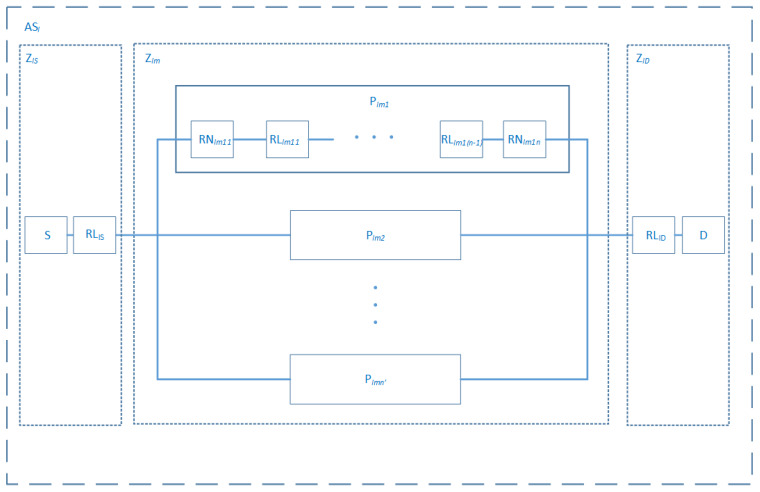
Core Network RBD model.

Routing and identifying smart objects in the network are the main tasks of the Core Network layer. In the core network layer, data can reach the same destination following different paths. The best path selection is based on distributed algorithms deployed on scattered nodes, namely routers. Paradigms like link state or vector-distance, are upon which routing algorithms, or routing protocols, run and generate routing tables. Furthermore, we can say that this layer also deals with routing in sensor networks. Nodes are subject to hardware and software failures, while links can fail, for example, due to interference, noise, or inappropriate environmental conditions. Foremost, a wide network of routers, like the Internet, can be divided into several Autonomous Systems (AS), with a given entity controlling each of them and running Internal Gateway Protocol (IGP). Two or more AS are connected via the External Gateway Protocol. In turn, each AS is composed of zones. These can provide several paths to reach the same destination node. These are a composition of Nodes and links in a serial pattern. We conclude that modeling paths with RBD is the cornerstone from which the whole system can be modeled adopting the bottom-top approach paradigm. All of these give rise to the following model in Figure 10. A possible path, linking a source *S* to a destination *D*, is modeled with a system composed of components in a serial pattern. Components are, respectively, Routing Nodes $\left(RN_{i}\right)$ and Routing Links $\left(RL_{i}\right)$. Other paths that connect the same source and destination are represented according to the parallel pattern. Paths can be grouped into zones $\left(Z_{m}\right)$, which can also follow the same pattern as the one for path-level. All we must do is walking one-step towards high-level abstraction and considering paths as component forming the zone system. One more step can be made to reach one more high-level abstraction considering AS as a component and, the inter-network as the whole system. Relying on the decomposition axiom of system theory, we propose the reliability equation, only, for the first case from which we can deduce the equations of the other cases.

The structure function of path $P_{i}$ can be written as:

(24) $$\phi_{P_{l,m,i}}\left(X_{P_{l,m,i}}\left(t\right)\right)=\prod_{j=1}^{n}\prod_{k=1}^{n-1}X_{RN_{l,m,i,j}}\left(t\right)\times X_{RL_{l,m,i,k}}\left(t\right)$$

where

- $\phi_{P_{l,m,i}}$: Structure function of the path *i* in Zone *m* that belong to the AS *l*.
- $X_{P_{l,m,i}}\left(t\right)$: State vector of the path *i* in Zone *m* that belong to the AS *l*.
- $X_{RN_{l,m,i,j}}\left(t\right)$: State vector of a routing node *j* of the path *i* in Zone *m* that belong to the AS *l*.
- $X_{RL_{l,m,i,k}}\left(t\right)$: State vector of a routing link *k* of the path *i* in Zone *m* that belong to the AS *l*.

(25) $$R_{P_{l,m,i}}\left(t\right)=\prod_{j=1}^{n}\prod_{k=1}^{n-1}R_{RN_{l,m,i,j}}\left(t\right)\times R_{RL_{l,m,i,k}}\left(t\right)$$

- $R_{P_{l,m,i}}\left(t\right)$: Reliability function of the path *i* in Zone *m* that belong to the AS *l*.
- $R_{RN_{l,m,i,j}}\left(t\right)$: Reliability function of a routing node *j* of the path *i* in Zone *m* that belong to the AS *l*.
- $R_{RL_{l,m,i,k}}\left(t\right)$: Reliability function of a routing link *k* of the path *i* in Zone *m* that belong to the AS *l*.

For disjoint paths, we have the following equation relative to $Z_{l,m}$:

(26) $$R_{Z_{l,m}}\left(t\right)=\left(1-\prod_{i=1}^{n^{\prime }}\left(1-R_{P_{l,m,i}}\left(t\right)\right)\right)$$

where

- $R_{Z_{l,m}}\left(t\right)$: Reliability function of the Zone *m* that belong to the AS *l*.

Then the whole system model takes the following form:

(27) $$R_{AS_{l}}\left(t\right)=R_{l,S}\left(t\right)\times R_{RL_{l,S}}\left(t\right)\times R_{Z_{l,m}}\left(t\right)\times R_{RL_{l,D}}\left(t\right)\times R_{l,D}\left(t\right)$$

where

- $R_{l,S}\left(t\right)$: Reliability function of the source node that belong to the AS *l*.
- $R_{RL_{l,S}}\left(t\right)$: Reliability function of the direct link to the source node that belong to the AS *l*.
- $R_{RL_{l,D}}\left(t\right)$: Reliability function of the direct link to the destination node that belong to the AS *l*.
- $R_{l,D}\left(t\right)$: Reliability function of the destination node that belong to the AS *l*.

#### 4.2.5. Middleware Layer Model

This layer allows various smart objects coming from different manufacturers, using different data formats and making exchanges according to dissimilar protocols to communicate, as if they were the same entities. We call this faculty smart objects interoperability assurance. Another leading role of this layer is to give to the layer next immediately, namely the Application layer, an abstraction to the heterogeneity aspects of the lower ones. Consequently, IoT applications makers can focus more on their business logic rather than managing dissimilarities. It should be noted that middleware technologies considered in our work are those that fall under the umbrella of distributed systems. Then the use of cloud computing paradigm to host this middleware and to manage the significant underlying volumes of generated data is desired unless there is some legal prohibition.

**Figure 11:**
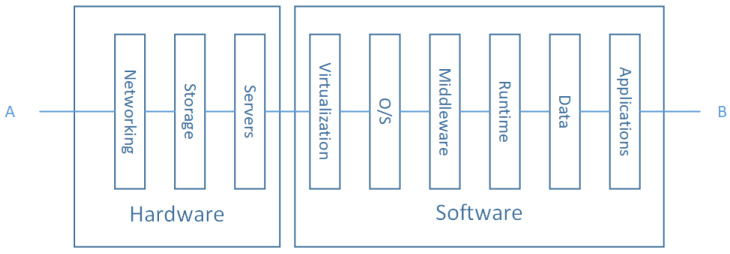
Middleware layer RBD model.

We propose in the present work to omit the unnecessary details that aim to distinguish the service models form one another and focus on the common features of the overall models. Given this assumption, let us merge all the above service models to be just one that is made of the nine layers that are common to each one of them. Which is more than enough from our adopted systemic approach point of view. Then our Reliability Block Diagram for the Middleware layer looks as shown in Figure 11.

The hardware part of the adopted model can be viewed as a graph, in which nodes are either Computing Nodes (CN), Storage Nodes (StN), Routing Nodes (RoN), Switching Nodes (SwN), or Security Nodes (SecN). Edges are links that connect the vertices. They can be from different nature such as fiber optics, copper wires, and so on. These statements bring us back to a similar situation of the Core Network Layer Model discussed above. The only differences are nodes flavors. Then, to model this part of the system, we can assume a path-based model where all critical stakeholder nodes are juxtaposed in serial patterns.

Alternative paths can be organized in a parallel pattern in the first approximation. More detailed study of Storage Area Network (SAN) reliability can be found in [39]. Although the Hardware part is crucial for reliable services, the software one has the same level of significance even more in some situations. In addition, some software properties make it unique. Namely, most, if not all, software used in the Middleware Layer are constellated around Big Data architecture styles because of the Volume, the Variety and the Velocity diversity of the generated data by the IoT devices. On the other hand, these architecture styles continue to change permanently to meet the maximum of the quality attributes. This makes software architecture views dynamic and evolvable. The most used architecture in IoT cloud platform is the so-called “Lambda” architecture besides the publish/subscribe style [40,41].

**Figure 12:**
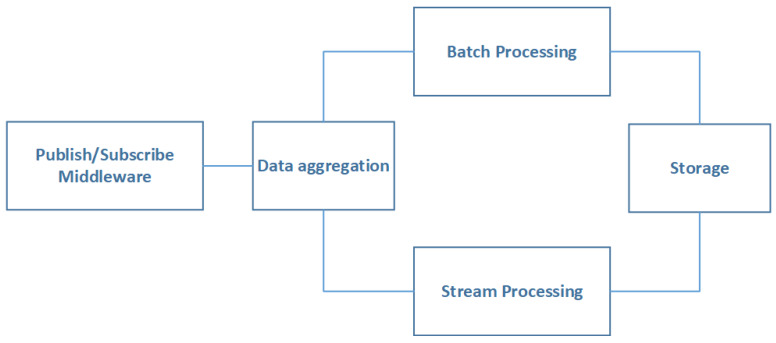
Topology view of Lambda based architecture.

The software layer in Figure 11 is made from virtualization, OS, Middleware, Data, and application layer. Whatever layer fails the software subsystem fails. The later subsystems can be analyzed more deeply considering the architectural styles building them. In this context, we can refer the works in [42] which propose to use a solution based on RBDs at the software architecture level to evaluate their impacts on reliability assessments. In our case, we are adopting the most widely used architecture as said above which is based on the Lambda style. Figure 12 shows a topology view of Lambda based architecture.

**Figure 13:**
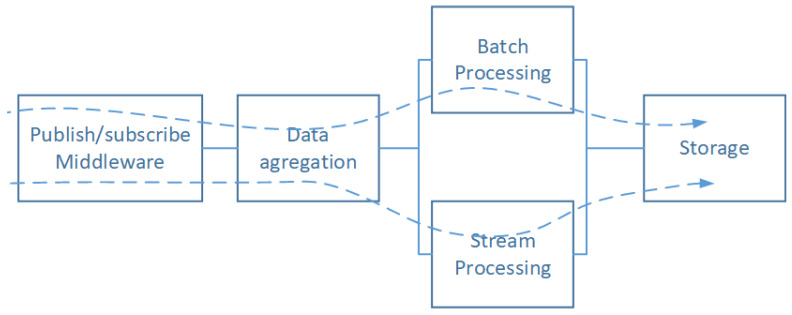
Lambda based architecture RBD model.

The topology in Figure 12 can be further broken down into two significant subsystems; each one of them is a possible path leading to the storage module. The first path relies on the flow of data that is processed by the Batch Processing (BP) subsystem. The second one deals with data that need to be processed with respect to the real-time constraint. The above assumptions give the result showed in Figure 13.

The components of the first subsystem, corresponding to the first path, are Publish/Subscribe (PS), Data Aggregation (DA), Batch Processing (BP), and the Storage Subsystem (St), which, are organized in a serial pattern. The second one is the same as the first one except for the Batch Processing subsystem that is replaced by Stream Processing subsystems (SP). It should be noted that all the blocks could be replicated to achieve scalability, which gives rise to parallel subsystems (children) of the same nature of the one to be scaled (parent). To simplify the proposed model, we assume that all subsystems are powerful enough to process large volume of data flows without the addition of any parallel component.

(28) $$R_{\lambda }\left(t\right)=R_{PS}\left(t\right)\times R_{DA}\left(t\right)\times \left(1-\left(1-R_{BP}\left(t\right)\right)\times \left(1-R_{SP}\left(t\right)\right)\right)\times R_{St}\left(t\right)$$

where

- $R_{\lambda }\left(t\right)$: Reliability function of λ-based architecture.
- $R_{PS}\left(t\right)$: Reliability function of the Publish/Subscribe subsystem.
- $R_{DA}\left(t\right)$: Reliability function of the Data Aggregation subsystem.
- $R_{BP}\left(t\right)$: Reliability function of the Batch Processing subsystem.
- $R_{SP}\left(t\right)$: Reliability function of the Stream Processing subsystem.
- $R_{St}\left(t\right)$: Reliability function of the Storage subsystem.

### 4.3. Reliability Framework of IoT Systems

**Figure 14:**
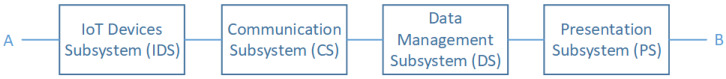
Framework of IoT End-to-End system RBD model.

In general, an End-to-End IoT system is made of similar blocks to those in Figure 14. Each block describes a given consistent subsystem that was already studied in the previous sections. The first one is the IoT Devices Subsystem noted (IDS) which was studied in Section 4.2.2 and Section 4.2.3. The second block is the Communication Subsystem (CS) which summarizes the serial association of the (AN) and the (CN) subsystems studied in Section 4.2.3 and Section 4.2.4. Data Management Subsystem (DS) and Presentation Subsystem (PS) are treated in Section 4.2.5, which deal with Middleware and Application Layers. Based on Figure 14, these subsystems follow a serial pattern. Then a global reliability equation can be expressed as:

(29) $$R_{GS}\left(t\right)=R_{IDS}\left(t\right)\times R_{CS}\left(t\right)\times R_{DS}\left(t\right)\times R_{PS}\left(t\right)$$

## 5. Framework Deployment Flow Chart

**Figure 15:**
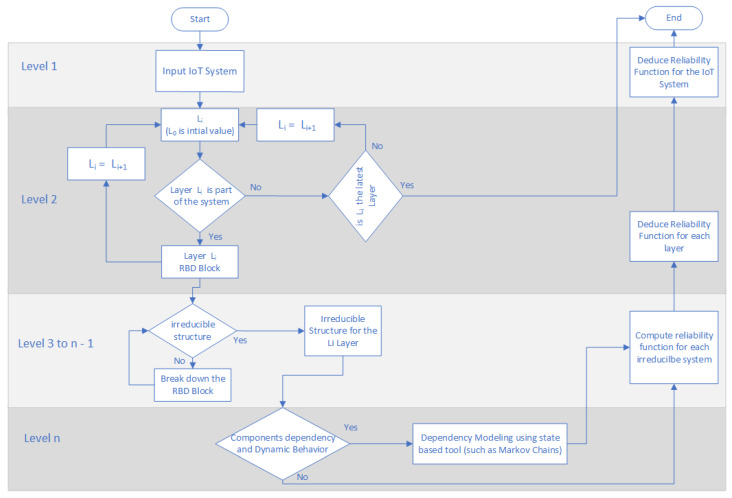
Framework deployment flow chart.

We propose the flow chart in the Figure 15 as a roadmap for implementing the proposed framework. The framework user may start defining the targeted system. The considered system will have implemented all or some of the layers in the adopted architecture. A layer is denoted $L_{i}$ with *i* an index of the architectural layers which are the Perception layer, Access Network layer, Core Network layer, Middleware layer, and Application layer. The considered layers are broken down until achieving an irreducible structure. Different methods for analyzing the reliability, such as Markov Chains, could be incubated by the framework. Finaly we adopt a bottom-up approach to assess the reliability of the whole System. This chart highlights that the proposed framework ensures scalability thanks to implementing hierarchy. Moreover, loops are internal to each level which reduce time complexity.

## 6. Numerical Investigation

We propose to study the behavior of a simplified architecture model according to some availability Targeted value. Furthermore, and for simplification, we propose(R(t)=exp(−λt)) the exponential distribution as a reliability law for all the random variables of all systems components. Indeed, the choice of the exponential distribution is justified by the fact that all the proposed system models in this work are built from a high-level abstraction process covering those systems’ high complexity. In [38,43], we can find that for some complex systems that operate for a significant amount of time, the exponential distribution plays a prominent role. Moreover, as a hypothesis, we consider that the reliability of both Data Management and Presentation Subsystems are identical to the identity.

### 6.1. Investigated Scenario

We consider in this section a scenario in which targeted availability is 90% within mission time. The corresponding λ parameter value is 1.203 × 10^−5^ , given the adopted exponential distribution, in a mission with 8760 h duration.

The equation to be considered in the simulation part of this work is a simplified form of that describing the model proposed in Section 4.3. In Section 4.3, we have introduced a model of IoT as a system made from four main subsystems ( Figure 14). Each of these subsystems has an analytical model. On one hand, we have adopted the *k*-out-of-*n* model for the IoT Devices Subsystem (IDS); on the other hand, we have proposed a parallel and series structures for the remaining subsystems as developed in the previous sections.

In our numeric investigation we propose to make the previous equations handier based on the following assumptions:

- The Access Network subsystem and the Core Network are considered the same from the reliability diagram point of view. The reason for this is that the path of the data can be one of those (parallel structure) that are made of Network Technologies nodes and Core Network nodes, which are in series.
- We have fixed the reliability function to be one for the Middleware and the application subsystems which correspond to (DS) and (PS) in Figure 14. The motivation behind this assumption is that the study of these subsystems cannot be included in this work because of their complexity and their specificity (Software case).

Given the pre-cited assumptions, we consider the following equation:

(30) $$R_{GS}\left(t\right)=R_{IDS}\left(t\right)\times R_{CS}\left(t\right)$$

which leads to the following numerical simplified model:

(31) RGS(ti)=(∑y=knnyR(ti)y×(1−R(ti)n−y)×(1−∏j=1n′(1−Rj(ti)))∏j=1n‘‘Rj(ti)

where:

(32) $$R\left(t_{i}\right)=R_{j}\left(t_{i}\right)=\exp\left(-\lambda t_{i}\right)$$

### 6.2. Results Interpretation

**Figure 16:**
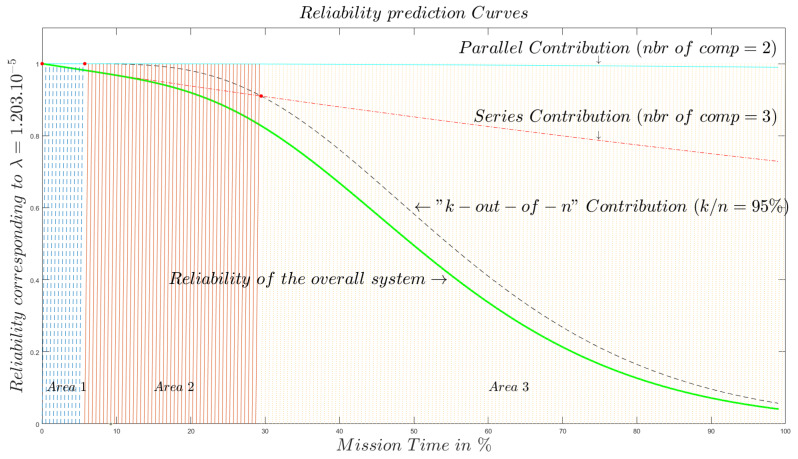
Reliability behavior of an End-to-End IoT system with k/n=95%.

Figure 16 shows four curves corresponding to the contribution of the perception subsystem modeled, as discussed previously, by a *k*-out-of-*n* system.

The contribution of the network subsystem which is modeled by the parallel components that represent success paths instances. The series contribution reflects the fact that the different main subsystems are mount in a series pattern. Finally, the fourth curve shows the evolution of the overall system reliability. The other subsystems are assumed to have reliability equal to the identity.

First, the four curves in Figure 16 show that every subsystem bloc brings a contribution that distinguishes it from the others. In addition, the curve of the overall system follows the one of the *k*-out-of-*n* subsystems. We observe that the parallel and series components contribute with the same shape, but the series ones decrease very quickly comparing to the parallel ones. Furthermore, our proposed model tells that the mission time can be divided into three areas. The first one is the region in which all components of the overall system are to be considered the same from a reliability prediction point of view. The second area (area 2) begins when the *k*-out-of-*n* subsystem reliability begins to diverge from one of the parallel subsystems while remaining more reliable than serial subsystem. The third one shows the fact that, in this region, more attention needs to be taken to the IoT Device Subsystem (IDS) compared to the other parts.

**Figure 17:**
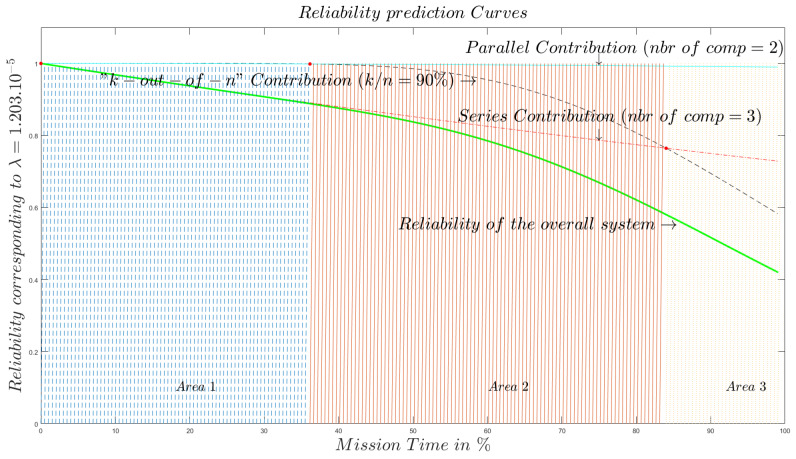
Reliability behavior of an End-to-End IoT system with k/n=90%.

The curves in Figure 17 are of the same nature as those in Figure 16. The only difference is the ratio of components needed to function in order that the *k*-out-of-*n* subsystem is functioning. In Figure 16 we assumed a ratio of 95% of working components for the *k*-out-of-*n* system to work. In Figure 17 the ratio is 90%. We can see that, for our proposed model, 5% of the difference in the ratio value of the perception subsystem implies considerable changes in terms of the size of the area.

**Figure 18:**
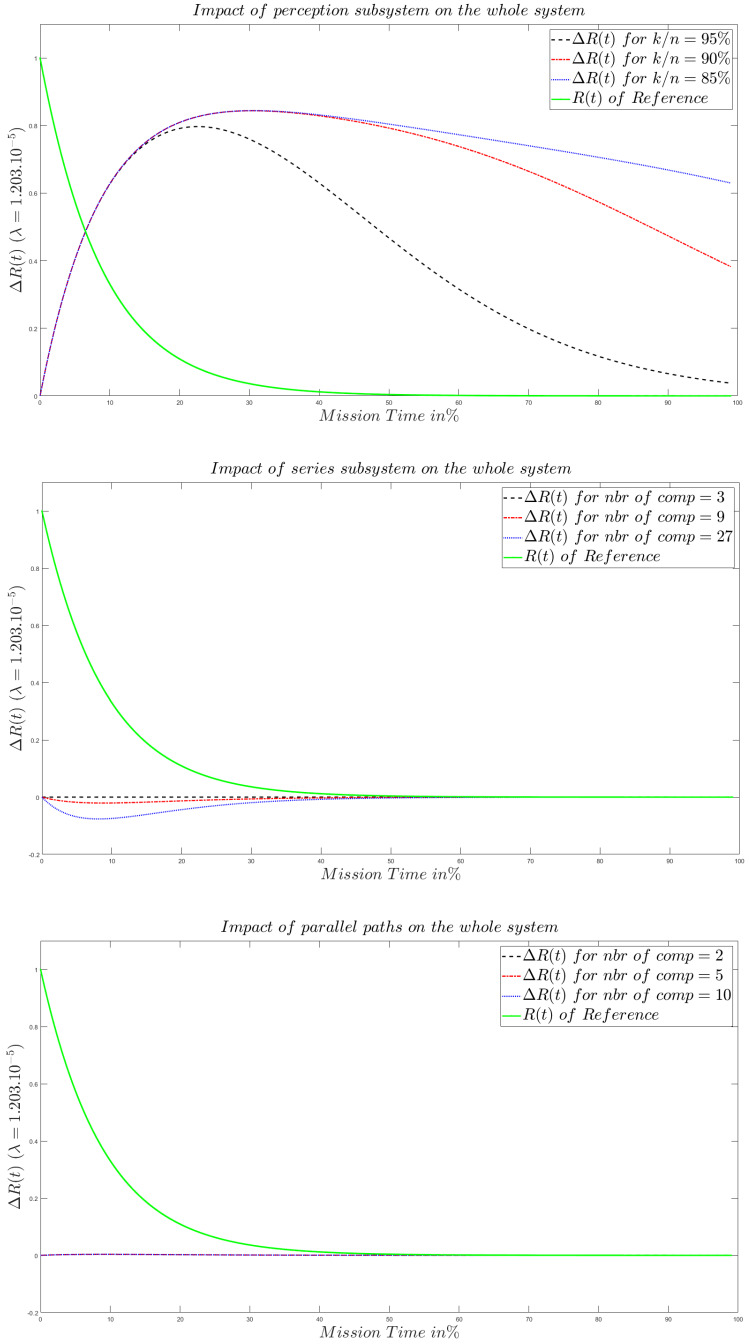
The impact of each structure on the whole system reliability behavior.

The impact of each structure on the overall system is studied from the curves in Figure 18. To carry out our study, we propose to compare the studied system w.r.t a reference system. This system is chosen to be the simplest one. Namely, three blocks in a series pattern without any parallel pattern redundancy with a system of *k*-out-of-*n* equal to 100%. A more complex system will be seen as a perturbation of this reference system. In Figure 18 we depict the difference between this perturbation and the curve of the reference system. As shown in Figure 18, the number of components that contribute to parallel and series patterns does not matter as the one of the *k*-out-of-*n* systems. Another thing to mention is the fact that in our context the reliability of the last one is the same until reaching some threshold of divergence.

All the above sayings lead us to conclude that, in our model with the respect of the previously specified conditions, the *k*-out-of-*n* subsystem decides the whole system reliability behavior and appears as an upper limit to the resulting reliability. In other terms, the perception layer plays the primary role to decide if an IoT system is reliable or not. In the other hand, the series pattern contribution is to decrease the reliability of the overall system when the number of the underlying components increases. The parallel pattern does not bring any significant contribution, according to the adopted model, to the reliability of the whole system as soon as the number of the components in parallel exceeds two.

## 7. Conclusions

The adoption of a systemic approach to architecting an End-to-End IoT system gave us a new opportunity to use existing formalisms. We have chosen the RBD paradigm to build our proposed model of an End-to-End IoT system. This brought us a tool to split the whole system into subsystems that can cohabit with each other. We mathematically modeled each subsystem based on context abstraction level. We arranged the resulting equations according to the adopted RBD model to give rise to a global model that predicts the reliability behavior for an End-to-End IoT system. Expressiveness, flexibility, time complexity, and hierarchy are the strengths of our proposed framework. We developed a flowchart to guide the framework user in the implementation process. Likewise, this flow chart revealed that the established framework granted scalability because of its hierarchical nature. The numerical results reported that the perception layer is the layer which impacting the most the reliability of the whole system. In addition, splitting mission time into remarkable areas, the obtained results revealed that our work can give rise to other ones related to the maintenance optimization of an End-to-End IoT system and the building of novel approaches to design IoT networks based on reliability criteria, to mentioning just a few.

## Figures and Tables

**Table 1:** Comparison of some reliability assessment methods.

Methods & Techniques	Preferred Step(s)	Benefits	Limitations	Ref
Failure Mode Analyses (FMMA, FMEA, and FMECA)	Design	Comprehensive Implementation	Scoring requires consensus	[28]
Tree Analyses (FTA, ETA, and BBN)	Design, Implementation	Events interdependencies are represented as a graph. Helpful for decision making	Heavy for high level granular system	[29,30,31,32]
RBD	Design	Design Optimization and uncertainties consideration	demanding in terms of computational resources	[19]
Hazard Analyses	Design, Operation	Hazards structuration Specifies operational risk factors	Heavy documentation Well-defined targeted system	[33]
Markov Chain	Operation, Maintenance	Used to model dynamic problems and maintenance processes	demanding in terms of computational resources	[34]
Monte Carlo Simulation	Operation, Maintenance	Straightforward Implementation Helpful for decision making	Computational resources consuming	[35]

## References

[1] Zhao K., Ge L. A survey on the internet of things security. Proceedings of the 2013 Ninth International Conference on Computational Intelligence and Security.

[2] Zhang Z.K., Cho M.C.Y., Wang C.W., Hsu C.W., Chen C.K., Shieh S. IoT security: Ongoing challenges and research opportunities. Proceedings of the 2014 IEEE 7th International Conference on Service-Oriented Computing and Applications.

[3] Palattella M.R., Accettura N., Vilajosana X., Watteyne T., Grieco L.A., Boggia G., Dohler M. (2013). Standardized protocol stack for the internet of (important) things. IEEE Commun. Surv. Tutor..

[4] Meddeb A. (2016). Internet of things standards: Who stands out from the crowd?. IEEE Commun. Mag..

[5] Ghayvat H., Mukhopadhyay S., Gui X., Suryadevara N. (2015). WSN-and IOT-based smart homes and their extension to smart buildings. Sensors.

[6] Zanella A., Bui N., Castellani A., Vangelista L., Zorzi M. (2014). Internet of things for smart cities. IEEE Internet Things J..

[7] Koulali M.A., Koulali S., Tembine H., Kobbane A. (2018). Industrial Internet of Things-Based Prognostic Health Management: A Mean-Field Stochastic Game Approach. IEEE Access.

[8] Roblek V., Meško M., Krapež A. (2016). A complex view of industry 4.0. Sage Open.

[9] Wollschlaeger M., Sauter T., Jasperneite J. (2017). The future of industrial communication: Automation networks in the era of the internet of things and industry 4.0. IEEE Ind. Electron. Mag..

[10] Hassanalieragh M., Page A., Soyata T., Sharma G., Aktas M., Mateos G., Kantarci B., Andreescu S. Health monitoring and management using Internet-of-Things (IoT) sensing with cloud-based processing: Opportunities and challenges. Proceedings of the 2015 IEEE International Conference on Services Computing.

[11] Wan J., Chen M., Xia F., Di L., Zhou K. (2013). From machine-to-machine communications towards cyber-physical systems. Comput. Sci. Inf. Syst..

[12] Khan R., Khan S.U., Zaheer R., Khan S. Future internet: The internet of things architecture, possible applications and key challenges. Proceedings of the 2012 10th International Conference on Frontiers of Information Technology.

[13] Hanes D., Salgueiro G., Grossetete P., Barton R., Henry J. (2017). IoT fundamentals: Networking Technologies, Protocols, and Use Cases for the Internet of Things.

[14] Lea P. (2018). Internet of Things for Architects: Architecting IoT Solutions by Implementing Sensors, Communication Infrastructure, Edge Computing, Analytics, and Security.

[15] Hassan Q.F. (2018). Internet of Things A to Z: Technologies and Applications.

[16] Mongiello M., Patrono L., Di Noia T., Nocera F., Parchitelli A., Sergi I., Rametta P. A complex event processing based smart aid system for fire and danger management. Proceedings of the 2017 7th IEEE International Workshop on Advances in Sensors and Interfaces (IWASI).

[17] Poy H.M., Duffy B. (2014). A cloud-enabled building and fire emergency evacuation application. IEEE Cloud Comput..

[18] Arnett J. (1990). Jet Propulsion Laboratory, Reliability Analyses Handbook.

[19] Dâmaso A., Rosa N., Maciel P. (2014). Reliability of wireless sensor networks. Sensors.

[20] Deif D., Gadallah Y. (2017). A comprehensive wireless sensor network reliability metric for critical Internet of Things applications. EURASIP J. Wirel. Commun. Netw..

[21] Kanabar M.G., Sidhu T.S. Reliability and availability analysis of IEC 61850 based substation communication architectures. Proceedings of the 2009 IEEE Power and Energy Society General Meeting, PES ’09.

[22] Hai Y., Yue Y., Yao Q., Yin H. Analysis on the reliability of wide area protection communication system. Proceedings of the International Conference on Communication Technology Proceedings, ICCT.

[23] Wei B., Lin C., Kong X. Dependability modeling and analysis for the virtual data center of cloud computing. Proceedings of the 2011 IEEE International Conference on High Performance Computing and Communications.

[24] Nguyen T.A., Min D., Choi E. (2020). A hierarchical modeling and analysis framework for availability and security quantification of IoT infrastructures. Electronics.

[25] Botta A., De Donato W., Persico V., Pescapé A. On the integration of cloud computing and internet of things. Proceedings of the 2014 International Conference on Future Internet of Things and Cloud.

[26] BSI (1991). BS 4778-3.1:1991, Quality Vocabulary. Availability, Reliability and Maintainability Terms. Guide to Concepts and Related Definitions.

[27] Rausand M., Høyland A. (2004). System Reliability Theory: Models, Statistical Methods, and Application.

[28] Ashley L., Armitage G. (2010). Failure mode and effects analysis: An empirical comparison of failure mode scoring procedures. J. Patient Saf..

[29] Wilson A.G., Huzurbazar A.V. (2007). Bayesian networks for multilevel system reliability. Reliab. Eng. Syst. Saf..

[30] Neil M., Littlewood B., Fenton N. (1996). Applying Bayesian belief networks to system dependability assessment. Safety-Critical Systems: The Convergence of High Tech and Human Factors.

[31] Torres-Toledano J.G., Sucar L.E. (1998). Bayesian networks for reliability analysis of complex systems. Ibero-American Conference on Artificial Intelligence.

[32] Khakzad N., Khan F., Amyotte P. (2011). Safety analysis in process facilities: Comparison of fault tree and Bayesian network approaches. Reliab. Eng. Syst. Saf..

[33] Dunjó J., Fthenakis V., Vílchez J.A., Arnaldos J. (2010). Hazard and operability (HAZOP) analysis. A literature review. J. Hazard. Mater..

[34] Li L., Jin Z., Li G., Zheng L., Wei Q. Modeling and analyzing the reliability and cost of service composition in the IoT: A probabilistic approach. Proceedings of the 2012 IEEE 19th International Conference on Web Services.

[35] Gertsbakh I.B., Shpungin Y. (2016). Models of Network Reliability: Analysis, Combinatorics, and Monte Carlo.

[36] Green J. (2014). IoT Reference Model. https://www.iotwf.com/resources/72.

[37] ITU (2013). Recommendation ITU-T Y.2060: Overview of the Internet of Things.

[38] Dharmaraja S., Jindal V., Varshney U. (2008). Reliability and survivability analysis for UMTS networks: An analytical approach. IEEE Trans. Netw. Serv. Manag..

[39] Xing L., Tannous M., Vokkarane V.M., Wang H., Guo J. (2017). Reliability Modeling of Mesh Storage Area Networks for Internet of Things. IEEE Internet Things J..

[40] Marz N., Warren J. (2015). Big Data: Principles and Best Practices of Scalable Real-Time Data Systems.

[41] Tarkoma S. (2012). Publish/Subscribe Systems: Design and Principles.

[42] Abd-Allah A. (1997). Extending reliability block diagrams to software architectures. System.

[43] Barlow R.E., Proschan F. (1996). Mathematical theory of Reliability.

